# Diagnosis and differentiation of mature cystic teratoma of pancreas from its mimics: A case report

**DOI:** 10.1097/MD.0000000000023267

**Published:** 2020-11-20

**Authors:** Xin he Zhou, Ji Kong Ma, Bimbadhar Valluru, Kalyan Sharma, Ling Liu, Jin Bo Hu

**Affiliations:** a Department of Radiology, the First Affiliated Hospital of Dali University; b Department of Nephrology, Dali Bai Autonomous Prefecture People's Hospital Dali 671000, China.

**Keywords:** dermoid cyst, differential diagnosis, magnetic resonance imaging diagnosis, pancreatic cystic teratoma, pancreatic neoplasm

## Abstract

**Introduction:**

Mature cystic teratoma originating in the pancreas is very unusual, often observed as an incidental finding during routine examinations or recognized perioperatively as the patients present with very unspecific clinical symptoms. The confirmatory diagnosis of a pancreatic cystic teratoma is generally made by histopathology after surgical excision. So, the preoperative diagnosis is very challenging, especially differentiation from the other pancreatic pathologies.

**Patient concerns:**

A 23-year-old woman was admitted to our hospital with a complaint of mild grade periumbilical abdominal pain. A pancreatic mass was revealed on a preliminary abdominal ultrasound examination. Her medical history was unremarkable with no long-standing illness or malignancy.

**Diagnosis:**

Mature cystic teratoma in the head of the pancreas.

**Interventions:**

Roux-enY choledochojejunostomy with gastrojejunostomy was performed, excising the tumor from the pancreatic head.

**Outcomes:**

The postoperative course was uneventful; the patient was asymptomatic and has no evidence of recurrence on a 2-year follow up.

**Conclusions:**

Pancreatic cystic teratoma is a benign, well-differentiated, and extremely rare congenital tumor. MRI is the choice of imaging modality and phase-GRE or fat suppression is the best technique for pre-operative diagnosis.

## 1 Introduction

Teratoma is a congenital tumor that originates from totipotent stem cells which are derived from all three germ layers namely ectoderm, endoderm, and mesoderm. They probably originate from the aberrant germ cells which are arrested during migration to the gonads in the early embryonic life. They usually emerge along the midline of the body and can be classified as mature, immature, and monodermal or highly specialized types. Mature type teratoma is benign and immature types are malignant. Moreover, mature type teratoma is further subdivided into a cystic and solid types, out of which, the cystic type is most commonly seen in the ovary and testes. However, a teratoma in the pancreas is an extremely unusual primary site of origin.^[1]^ Due to their better differentiation and preponderance towards the ectodermal germ layer, they are also referred to as dermoid cyst. The emergence of a mature pancreatic cystic teratoma was first described by Kerr in 1918.^[2]^ We report the rare case of a primary pancreatic dermoid cyst in the head of the pancreas in a 23-year-old female patient presenting with very unusual non-specific symptoms.

## 2 Case report

### 2.1 Patient information

A 23-year-old female patient presented to the out-patient department with a history of intermittent mild grade periumbilical abdominal pain for one day and she was put under observation considering being an acute abdomen. The pain was not alleviated, she was admitted into our hospital for further evaluation and management. Her physical examination and vitals were unremarkable. She did not have any history of long-standing illness and malignancy. There is no history of any abdominal surgeries or trauma. Preliminary ultrasonography (USG) of the abdomen was recommended.

## 3 Imaging examination

All the materials and methods are carried out according to the guidelines and regulations set by our institution. Based on the provisional findings on USG, a complete radiological assessment was recommended with non-contrast computed tomography (NCCT/Plain) and contrast CT (CCT), followed by an abdominal Magnetic Resonance Imaging (MRI), both plain and contrast-enhanced sequences were obtained respectively.

## 4 Imaging manifestations

**Figure 1:**
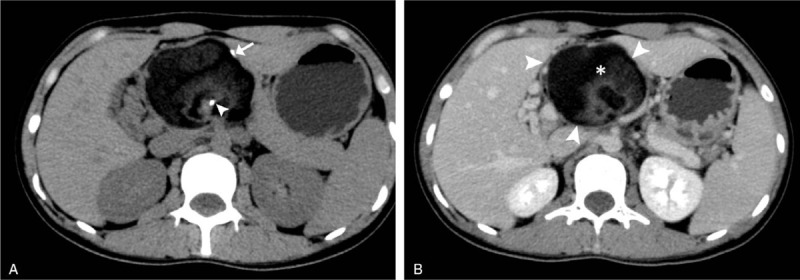
(A) Plain CT showing partially calcified capsule (arrow) with calcification within the mass (arrowhead). (B) Contrast-enhanced CT showing a large heterogeneous mass with slight peripheral enhancement (arrowheads) in the head of the pancreas with a large inhomogeneous area (asterisk).

**Figure 2:**
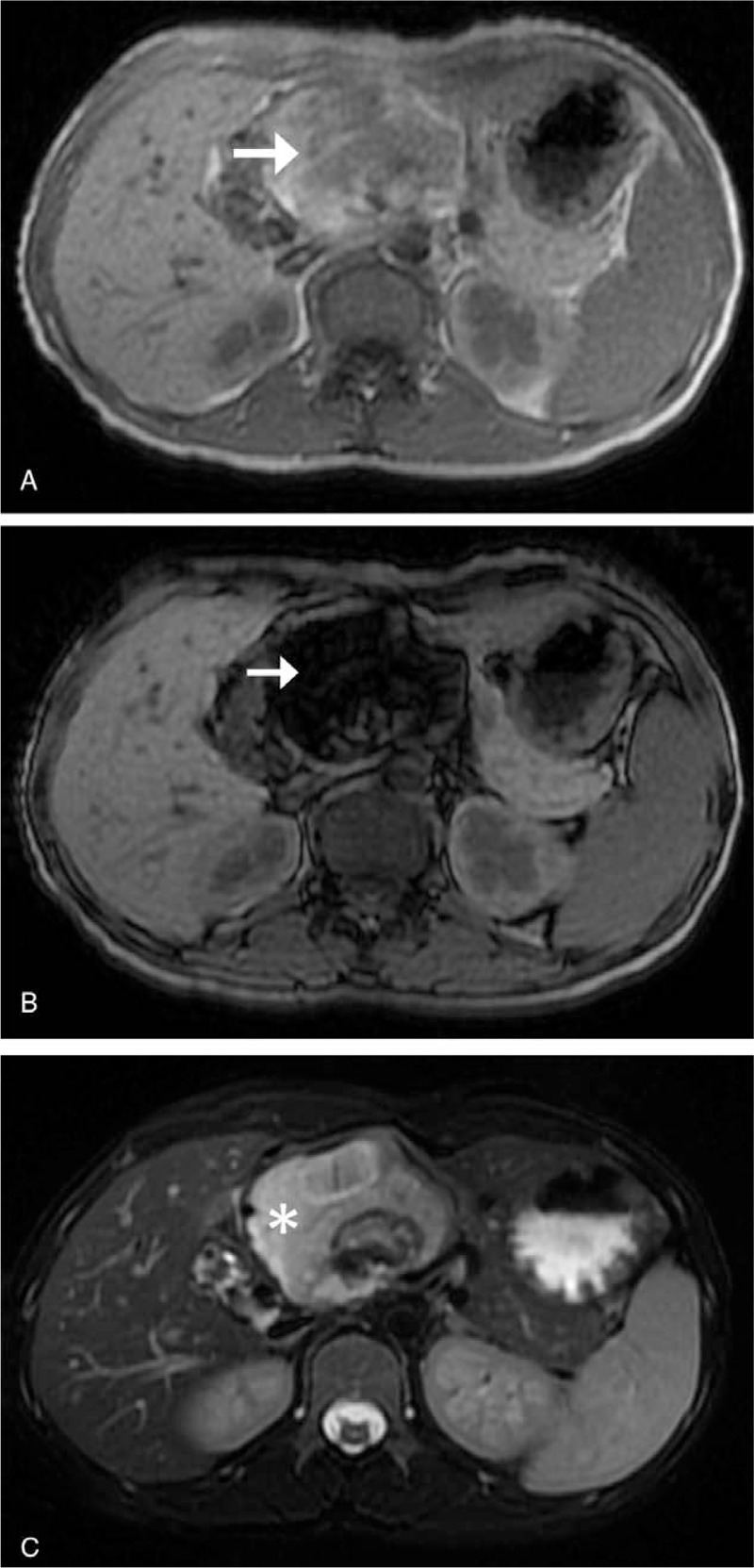
Magnetic resonance imaging (MRI) (A) In-phase sequence showing heterogeneous high signal intensity mass (arrow) in the head of the pancreas. (B) Out-of-phase sequence showing a drop out (low) signal (arrow). (C) T2-fs showing a heterogeneous high signal.

Abdominal USG revealed a well-defined mass of size 8.3 x 7.4 cm along the head of the pancreas that showed predominant echogenicity within a thin capsule. Non-contrast abdominal CT scan revealed a hypodense mass measuring about 8.3 x 7.4 x 7.1 cm, arising from the head of the pancreas with evident relative mass effect on the surrounding tissues. There were no other corresponding signs like fat stranding and effusions indicative of pancreatitis or no evidence of pancreatic and common bile ducts dilatation. A solid fat component and the focal calcified cystic wall were noted (Fig. 1A). On contrast-enhanced CT, the mass demonstrated inhomogeneous hypoattenuating pattern with mild enhancement of the wall (Fig. 1B). MRI revealed a protein-rich fluid component in the cystic mass and revealed a heterogeneous iso to high intense signal followed by low- intense signal on T1- weighted imaging (T1WI) in keeping with loss of signal intensity between in-phase (IP) and out-phase (OOP) sequences (Fig. 2A-B), as well as a heterogeneous low-intense signal on T2-weighted imaging with Fat suppression (T2WI-Fs) collectively suggesting the presence of macroscopic fat in the lesion (Fig. 2C). Functional tests of the pancreas and liver were within normal limits. Tumor markers CEA (Carcinoembryonic antigen), AFP (Alfa-fetoprotein) in the serum were normal, yet CA (Carbohydrate Antigen)-125 was up to 86.14 U/mL (normal range <39 U/mL) and CA 19-9 was up to 54.54 U/mL (normal range <35 U/mL) suggesting significant elevation.

## 5 Histopathological results

**Figure 3:**
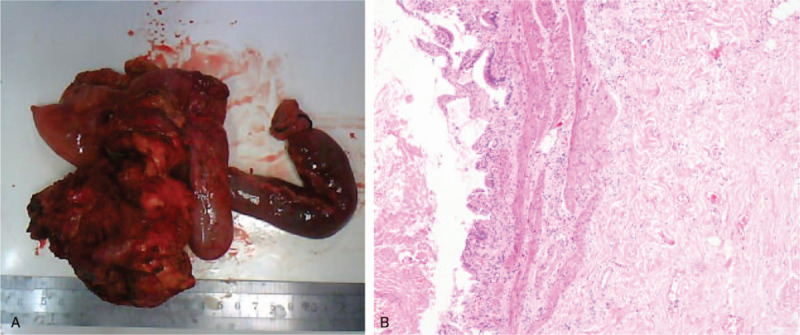
(A) Gross anatomical resected specimen of pancreatic teratoma. (B) Histopathological section of pancreatic teratoma stained with H&E stain (Hematoxylin and Eosin) ×40).

Intraoperatively, the tumor was seen to originate from the head of the pancreas and was adherent to the duodenum, common hepatic artery, and portal vein. Roux-en-Y choledochojejunostomy with gastrojejunostomy was performed, excising the tumor from the pancreatic head. An excised specimen sent for further histopathological examination revealed yellowish-white material, evident macroscopically suggestive of caseous necrosis (Fig. 3A). Microscopically, the cyst was lined by stratified epithelium, glandular epithelium, stroma with germinal centers, and surrounding abundant lymphoid infiltration (Fig. 3B). The postoperative course was uneventful and the patient was discharged on the 17th postoperative day with no further complications. The patient was asymptomatic and has no evidence of recurrence on a 2-year follow up.

## 6 Discussion

Teratoma is a congenital tumor arising from embryonic residues as germ cell tumors which are divided into three sub-types namely mature, immature, and monodermal or highly specialized. Most of the mature types are strictly regarded as well- differentiated benign lesions, so often referred to as dermoid cysts.^[1]^ Ovaries and testes are the most common sites of origin but can also be found at any site along the route of ectodermal cell migration, usually in the midline, such as the brain, cranium, mediastinum, omentum, retroperitoneum, and sacrococcygeal regions.^[2]^ However, a cystic teratoma in the pancreas is very rare, including this case, there are 51 cases reported in the literature to date.^[3]^ On reviewing the literature of all 50 cases, 54% were male and the remaining 46% were female suggesting a slight male predominance. However, the age groups were 4 months to 75 years old and the mean age group was 37.7 years old within both sexes. Most of the patients typically present with non-specific gastrointestinal symptoms like abdominal pain, nausea, vomiting, lower back pain, and weight loss, and sometimes with completely irrelevant symptoms.^[4]^

With varying rates of sensitivities and specificities, tumor markers like CA 19-9, CA 125, CA 72-4, and CEA have been useful in differentiating benign from malignant. A very high label of CA 125 and CA 19-9 will represent the malignant variety and a small increment from the normal range will represent a benign lesion.^[5]^ The present study also showed the slightly elevated label of both CA 19-9 and CA-125 which was 54.54 U/mL and 86.14 U/mL respectively. For the unremarkable routine laboratory results and clinical presentation, the diagnosis of mature teratoma mainly relies on imaging techniques such as abdominal ultrasonography (USG), Computed Tomography (CT), Magnetic Resonance Imaging (MRI). Moreover, the imaging appearance of the lesion depends on the proportions of tissue components such as the presence of fat, fat-fluid levels, and calcifications.^[6]^

Previous studies have suggested that the most common site was on the pancreatic body (35.6%), along with the head (33.3%) and followed by the tail (15.5%).^[3]^ The average size of the tumor was approximately 8 cm with a minimum and maximum size of 2.2 cm and 25 cm respectively.^[1,3]^ Given the unusual location, the pancreas, many are not diagnosed until much later in adulthood, despite originating in utero as congenital tumors.

On the abdominal USG, a clear margin without internal septations along with areas of high-fat content appear hyperechoic, whereas calcified tissues are seen as focal areas with posterior acoustic shadowing.^[4]^ Since the hyperechoic appearance of the regional fat component overlaps with other types of soft tissues, USG alone cannot be considered as the diagnostic tool.

On CT scans, they usually appear as a round hypodense lesion with a clear boundary and Hounsfield unit (HU) measuring around −20 HU to −140 HU, which could be due to intratumoral content mainly fat. The intratumoral content like fat, fat-fluid levels, and calcification are highly suggestive of a mature cystic variety of pancreatic teratoma.^[6]^ So, CT is considered as a sensitive technique and superior to USG for characterization, localization, as well as intratumoral detection of calcification or fat, can be evaluated very easily.

MRI, on the other hand, provides an excellent soft-tissue contrast resolution and is highly specific for detecting soft tissue lesions. T1WI in-phase (IP) and out-of-phase (OOP) imaging are very useful for the fat-containing lesions and to identify the disease states related to the presence of diffuse or focal steatosis in the liver, pancreas, adrenal glands, etc. Pancreatic lesions like teratoma have high signal intensity on T1WI and T2WI TSE (turbo spin-echo) images similar to that of subcutaneous fat.^[7]^ Some hemorrhagic lesions (containing methemoglobin) will also demonstrate similar imaging features. So, distinction by conventional MR imaging techniques will be difficult. Therefore, the use of techniques like phase-shift GRE or fat suppression is recommended in the differential diagnosis with the hyperintense signal in T1WI.^[8]^ For the differentiation, Phase-shift GRE sequences correspond to in-phase and out-of-phase with the same repetition time (TR) but with two different echo time (TE) values. Microscopic intralesional fat from water within the same voxel can be easily identified and differentiated by observing loss of signal intensity on the phase-shift GRE sequence which shows drop out signal (low signal intensity) on the out-phase images compared to the in-phase images. Thus, these techniques should be useful and recommended in the differential diagnosis of such lesions by MR imaging.

So, USG plays a very important role in early detection, plain CT, and MRI help in classifying these tumors, contrast-enhanced studies help in analyzing the intra-tumoral characteristics for complete pre-surgical evaluation. However, the imaging features of pancreatic teratoma are often found to be similar to the other pancreatic neoplasm which can be easily misdiagnosed.

Surgical excision is the principal method for the management of pancreatic cystic teratoma which is highly dependent on the location and size of the tumor. Tumor resection, pancreaticoduodenectomy or Roux-en-Y choledochojejunostomy, external cyst drainage, or cyst gastric anastomosis are feasible. However, in recent years, simple external drainage of cysts is no longer practiced because of recurrence and emergence of pancreatic fistula. Moreover, internal drainage of the cystic gastric anastomosis is not recommended as it requires a long-term follow-up. Based on our case study, it is convenient to consider that such cystic tumors do not show a malignant potential, so, it is also feasible to choose close observation, anticipating to further evaluate the nature of the tumor completely during follow-up.

## 7 Differential diagnosis

### 7.1 Pancreatic pseudocyst

They are the most common complications of acute/chronic pancreatitis and pancreatic trauma. The tumor predominantly occurs in the head of the pancreas. On CT, the presence of limited round or oval shape, well-circumscribed, a homogeneously hypo-attenuating lesion with thick septations, and usually surrounded by a well-defined enhancing margin favors the diagnosis of the pseudocyst.^[9]^ It often causes a remarkable indentation on the posterior wall of stomach or adjacent bowel loop, a extrinsic mass effect demonstrating a “pad sign”. A contrast study will demonstrate mild early enhancement of the wall. On MRI, the cysts demonstrate a homogenous low T1 signal intensity center and homogeneous high T2 signal intensity. However, when pseudocyst is complicated due to necrosis, hemorrhage, or infection it will demonstrate the heterogeneous signal intensity.^[10]^

### 7.2 Serous cystadenoma

Serous cystadenomas are common benign cystic neoplasms of the pancreas often located in the head of the pancreas. They are usually seen in older women over 60-year-old and uncommonly reported as malignant.^[11]^ CT scan typically demonstrates numerous small cysts, sometimes described as a ‘bunch of grapes’, seen in pancreatic head, measuring few millimeters to 2 cm. On MRI, T1WI demonstrates a typical low signal while T2WI shows a high signal if a mass contains cystic components and a low signal if fibrous scar.^[12]^ A fibrous central scar with or without a characteristic stellate pattern of calcifications is considered to be virtually pathognomonic for Serous Cystadenoma at CT or MR imaging.^[13]^

### 7.3 Mucinous cystadenoma

Mucinous cystadenomas often occur in middle-aged women, mainly in the body or tail of the pancreas with high malignant potential. On CT imaging, the lesion manifests as a single round, internal septa with an average size of 10 cm. The wall of the cyst is usually thick and eggshell calcifications may be seen.^[14]^ MRI imaging demonstrates heterogeneous high signal intensity on T1WI and homogenous high signal intensity on T2WI.^[15]^

### 7.4 Solid pseudo-papillary tumors (SPT)

SPT is an uncommon borderline or low-grade malignant potential tumor, which occurs mainly in women over 20 years old, and can arise anywhere in the pancreas.^[16]^ The abdominal CT shows large size solitary lesion, heterogeneous density mass with occasional.^[17]^ On MR, the papillary solid part T1WI appears as a low to iso-intensity and hyper to iso-intensity on T2WI moreover on contrast examination, the solid components display heterogeneous enhancement.^[18]^

### 7.5 Epidermoid cyst in the intrapancreatic accessory spleen (ECIPAS)

ECIPAS was first reported by Davidson et al^[19]^ in 1980. Since then only case reports have been reported. The age of onset varies from 12 to 70 years, with a slight female predominance. Racial factors have been associated with ECIPAS reported mostly in Asian races,^[20]^ occurring mostly in the tail of the pancreas with a diameter ranging from 1.4 cm to 15 cm. On CT plain scan, it shows a hypodensity in the cystic part, the solid part, and the cystic wall appeared as isodense or slight hyperdensity; on contrast-enhanced scanning, the cystic part does not enhance, but enhancement may be obvious along the solid part, especially along the cystic wall. On MRI, the cystic part shows a hypointense signal on T1WI, a hyperintense signal on T2WI, and a slightly high signal on DWI. The imaging features of the cyst are that the solid components of the cyst show similar enhancement with the adjacent splenic parenchyma on enhanced CT and magnetic resonance T1 images.^[21]^ The correct preoperative diagnosis of ECIPAS required sufficient enhancement of the respective splenic tissue around the cyst.^[22]^

### 7.6 Lymphoepithelial cysts (LECs)

LECs are one of the rare non-neoplastic true cysts of the pancreas, predominantly occurring in mostly in men that are aged 20 to 85 (average incidence around 55 years) with no specific clinical manifestations. CT features are slightly low or isodense solitary mass mostly in the body and tail of the pancreas with visible cystic walls and intralesional septations. They can also occur in or around the pancreas. Spotted calcification along the cystic walls, slight enhancement of the posterior cystic walls, and septations might be evident. There will be no dilation of the pancreatic duct. On MR, it is characterized by a high signal on T1WI and low signal on T2WI.^[23]^

### 7.7 Consent for publication

Written and informed consent was obtained from the patient for publication of this case report and accompanying images.

## 8 Conclusion

Mature cystic teratoma is also called a dermoid cyst of the pancreas. An ovary is the most common primary site and the pancreas is the rarest. A well-defined encapsulated mass with intralesional fat, fluid-fat, and calcifications are highly suggestive of mature teratoma. MRI is the choice of modality and phase-GRE or fat suppression is the best technique. Drop out a signal on the out-phase images compared to the in-phase images indicates intravoxel lipid. Radiologists should be familiar with this entity and include it into the differential diagnosis of cystic neoplasm of the pancreas.

## Author contributions

**Conceptualization:** Xin he Zhou.

**Data curation:** Xin he Zhou, Bimbadhar Valluru, Kalyan Sharma, Ling Liu.

**Formal analysis:** Xin he Zhou, Ji Kong Ma, Bimbadhar Valluru, Kalyan Sharma, Ling Liu, Jin Bo Hu.

**Investigation:** Xin he Zhou, Bimbadhar Valluru, Kalyan Sharma, Ling Liu, Jin Bo Hu.

**Methodology:** Xin he Zhou, Ji Kong Ma, Bimbadhar Valluru, Kalyan Sharma, Ling Liu, Jin Bo Hu.

**Project administration:** Ji Kong Ma, Ling Liu.

**Resources:** Bimbadhar Valluru, Ling Liu, Jin Bo Hu.

**Supervision:** Ji Kong Ma, Kalyan Sharma, Ling Liu, Jin Bo Hu.

**Validation:** Bimbadhar Valluru, Kalyan Sharma, Ling Liu, Jin Bo Hu.

**Visualization:** Bimbadhar Valluru, Kalyan Sharma, Ling Liu, Jin Bo Hu.

**Writing – original draft:** Xin he Zhou, Ji Kong Ma.

**Writing – review & editing:** Xin he Zhou, Ji Kong Ma, Bimbadhar Valluru, Kalyan Sharma, Ling Liu, Jin Bo Hu.
